# Coping With Water Shortage: An Update on the Role of K^+^, Cl^-^, and Water Membrane Transport Mechanisms on Drought Resistance

**DOI:** 10.3389/fpls.2019.01619

**Published:** 2019-12-20

**Authors:** Manuel Nieves-Cordones, Francisco García-Sánchez, Juan G. Pérez-Pérez, Jose M. Colmenero-Flores, Francisco Rubio, Miguel A. Rosales

**Affiliations:** ^1^Departamento de Nutrición Vegetal, Centro de Edafología y Biología Aplicada del Segura–CSIC, Murcia, Spain; ^2^Centro para el Desarrollo de la Agricultura Sostenible (CDAS), Instituto Valenciano de Investigaciones Agrarias (IVIA), Valencia, Spain; ^3^Instituto de Recursos Naturales y Agrobiología, Spanish National Research Council (CSIC), Sevilla, Spain

**Keywords:** drought stress, water deficit, plant, potassium, chloride, water transport, fertilizer

## Abstract

Drought is now recognized as the abiotic stress that causes most problems in agriculture, mainly due to the strong water demand from intensive culture and the effects of climate change, especially in arid/semi-arid areas. When plants suffer from water deficit (WD), a plethora of negative physiological alterations such as cell turgor loss, reduction of CO_2_ net assimilation rate, oxidative stress damage, and nutritional imbalances, among others, can lead to a decrease in the yield production and loss of commercial quality. Nutritional imbalances in plants grown under drought stress occur by decreasing water uptake and leaf transpiration, combined by alteration of nutrient uptake and long-distance transport processes. Plants try to counteract these effects by activating drought resistance mechanisms. Correct accumulation of salts and water constitutes an important portion of these mechanisms, in particular of those related to the cell osmotic adjustment and function of stomata. In recent years, molecular insights into the regulation of K^+^, Cl^-^, and water transport under drought have been gained. Therefore, this article brings an update on this topic. Moreover, agronomical practices that ameliorate drought symptoms of crops by improving nutrient homeostasis will also be presented.

## Introduction

Water is the essence of life. Plants, like the rest of living organisms, need water to complete their life cycle. Land plants have the additional handicap that they are anchored to the soil and rely on weather, soil, groundwater aquifers, and on their root growth capacity to ensure water availability. Moreover, land plants are not very efficient in the use of water since 95% of the water taken up by the plant is lost by transpiration (Kramer, 1983), making them quite vulnerable to water scarcity. This situation is very common since approximately one-third of the Earth’s land is arid or semi-arid and it is expected that this fraction increases due to climate change in the following decades (El-Beltagy and Madkour, 2012). In fact, drought is the abiotic stress with the biggest negative impact on the sustainability of agriculture and food security (Farooq et al., 2009). Drought strongly reduces plant growth and yield due to the loss of cell turgor, reduction of CO_2_ assimilation, oxidative stress, and nutritional imbalance among other effects (Farooq et al., 2009). Water deficit (WD) is defined as an imbalance between water supply and demand. WD can be originated around roots due to soil drying and around leaf cells due to low air humidity and high temperature (Tardieu et al., 2018). It is worth highlighting that in arid environments, which will be present in larger world areas in the future, both WD conditions occur at the same time and plant responses may be different under stress combination in comparison to single WD conditions. Thus, studies taking into account these aspects are of special relevance (Georgii et al., 2017). The response of crops to WD depends on the timescale considered (Tardieu et al., 2018). Short-term responses are related to the adjustment of stomatal conductance, water potential differences among tissues, hydraulic conductance (including that of xylem and of phloem), osmolyte content and turgor pressure and organ growth. Long-term responses are associated with crop cycle duration, grain abortion, root architecture, nutrient allocation (including total nonstructural carbohydrates), leaf/root phenological cycles, cell-dehydration tolerance mechanisms, and delayed senescence. Part of these responses aims at saving water such as stomatal closure (WD avoidance) whereas others aim at coping with the low water status such as osmoregulation (WD acclimation). WD is rapidly sensed by plant cells and significant advances in the identification of sensing mechanisms have been made in the last years. Reactive oxygen species and Ca^+2^ signaling enter the scene very early and allow shaping and propagation of the signal (Demidchik, 2018). When WD takes place at the soil level, a root-to-shoot transmission of the WD signal seems to be mediated to some extent by sulfate which precedes abscisic acid (ABA) synthesis in leaf tissues (Ernst et al., 2010; Malcheska et al., 2017; Batool et al., 2018). However, when WD is produced by low air humidity, this condition is perceived by guard cells which synthesize ABA and, then, stomata close (Bauer et al., 2013). Indeed, among plant hormones, ABA plays a major role to drought stress responses due to its involvement in crucial processes such as stomatal closure, root-to-shoot ion translocation, and modulation of root growth (Cram and Pitman, 1972; Pitman and Wellfare, 1978; Ma et al., 2018). Cross-talk between ABA and other hormones such as auxin, jasmonic acid, ethylene, and cytokinins has been reported (Tanaka et al., 2006; Benlloch-Gonzalez et al., 2010; Aleman et al., 2016; Rowe et al., 2016; Huang et al., 2018) which enormously increase the variety of hormonal signals taking part during drought stress.

Nutrient homeostasis is a key process for WD resistance. This is exemplified by the fact that nutrient deficiency symptoms are observed in plants under drought stress conditions and an increased input of nutrients helps in the mitigation of drought symptoms in plants (Waraich et al., 2011). Rapid osmotic adjustment in response to WD results from a fast accumulation of the inorganic ions such as K^+^ and Cl^–^ in the vacuole, allowing rapid recovery of the cell osmotic potential and maintenance of cell turgor (Shabala et al., 2000; Shabala and Lew, 2002). Given that K^+^ and Cl^–^ are not metabolically assimilated and due to their high availability, mobility, and very low molecular weight, these inorganic ions are very effective osmoregulatory molecules. Concomitant to K^+^ and Cl^-^ movements, cell hydraulics also plays a crucial role in the acclimation to the WD, since they allow the plant to adjust water flux and to set a cell water potential that is better suited for the water-limiting condition and to facilitate solute movements (Scharwies and Dinneny, 2019). Thus, it is not surprising that a significant part of the cellular responses to WD had ion homeostasis and water membrane transport as the target process (Jarzyniak and Jasiński, 2014). Consequently, cellular mechanisms and agricultural practices improving K^+^, Cl^-^, and water transport should be considered as potential targets to breed drought-tolerant crops. In recent years, significant progress in this topic has been made which will be summarized in the present mini-review. We would like to indicate that, unless stated, WD means hereafter root-applied WD since it is the most used WD stress in the papers we cite.

### Potassium, Chloride, and Water Membrane Transport Under Water Deficit Conditions

#### Potassium Membrane Transport

**Figure 1:**
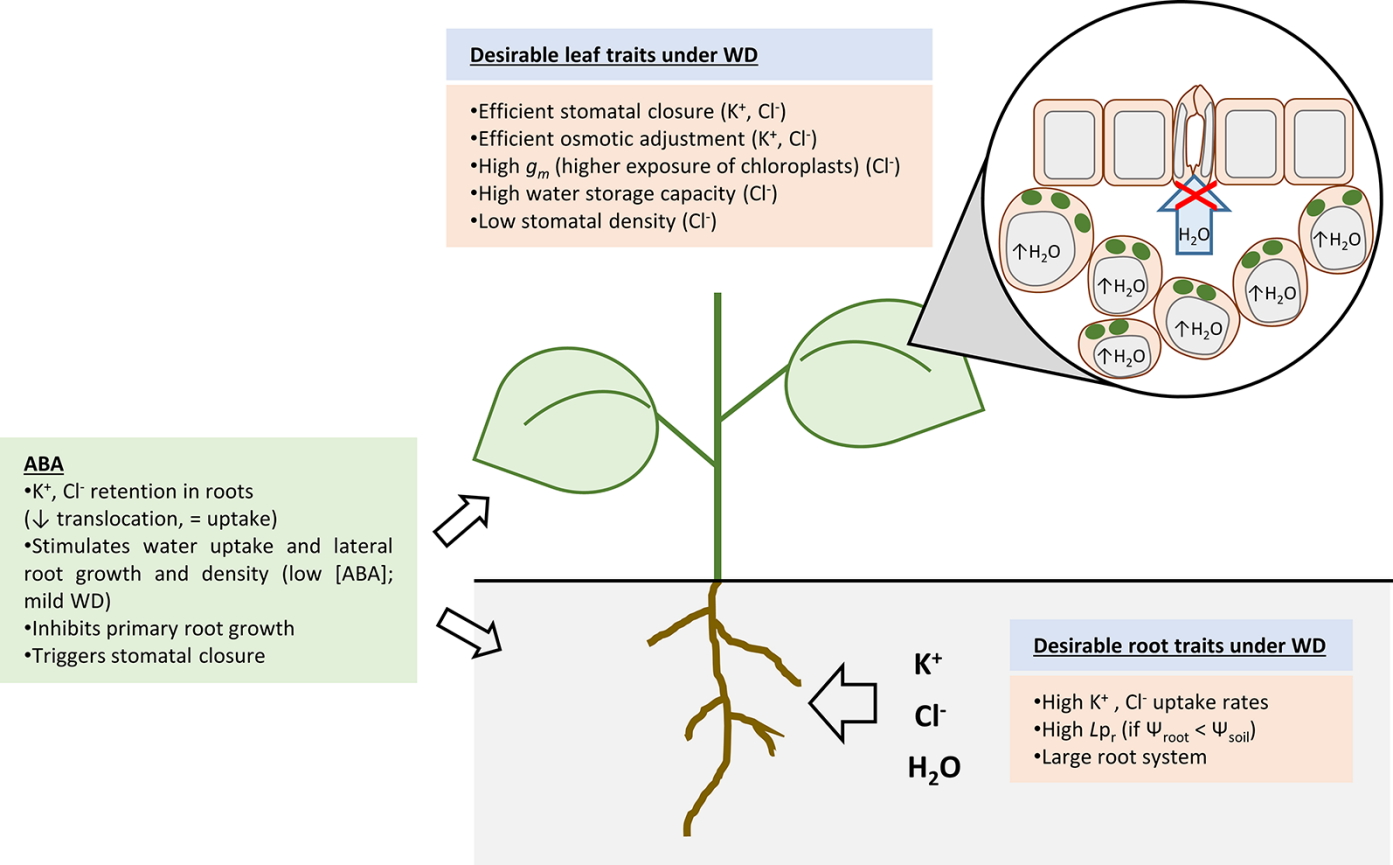
Overview of the main processes associated to water deficit (WD) resistance. Some of these processes aim at retaining water (drought avoidance, i.e., stomatal closure and root water uptake) while others aim at coping with reduced intracellular water content (drought tolerance, i.e., osmotic adjustment). It has been shown that high accumulation of K^+^ and Cl^-^ within the plant is beneficial for WD acclimation. Thus, high activity of K^+^ and Cl^-^ uptake systems and a large root system are desirable traits. Hydraulic conductivity of the root (*L*p_r_) under WD can be upregulated if the water potential of root cells has been readjusted in comparison to that of the soil. Thus, flow of water into the root can still occur. Abscisic acid plays a crucial role in WD responses as it triggers K^+^ and Cl^-^ retention in roots, changes in root system architecture (enhances lateral root growth and inhibits primary root growth), and stomatal closure. With respect to leaf tissues, K^+^ and Cl^-^ allow an efficient osmotic adjustment of leaf cells which is a key process to retain water within cells. Efficient stomatal closure prevents excessive water loss and is achieved by K^+^ and Cl^-^ release from guard cells. Cl^-^ has a specific beneficial effect in leaf cells by giving rise to larger cells (with higher water storage capacity), lower stomatal conductance (reduced stomatal density), and higher mesophyll conductance (*g* *_m_*) to CO_2_. Therefore, water use efficiency is increased under proper Cl^-^ nutrition.

Potassium is an essential macronutrient which fulfils critical functions in plants. For example, it enhances enzyme activity, protein synthesis, photosynthesis, phloem transport, charge balance, osmoregulation and takes part in stress signaling (Marschner, 2012; Shabala, 2017). Most, if not all, of these functions are negatively affected by WD (Farooq et al., 2009) and adequate K^+^ levels in the plant are important for mitigation of stress symptoms (Cakmak, 2005). K^+^ ions are highly mobile and are not metabolized. Therefore, membrane transport is the critical process to correctly distribute K^+^ among cells and compartments. The first step in K^+^ nutrition is the K^+^ absorption by root cells from the soil solution (**Figure 1**). At this level, drought has already a negative impact on K^+^ homeostasis since low soil moisture leads to reduced K^+^ mobility in the soil solution and thus reduced plant K^+^ uptake (Kuchenbuch et al., 1986). The impact on WD responses of manipulating root K^+^ transporters has been addressed in few studies. Rice K^+^ uptake transporters such as OsHAK1 and OsAKT1 have been shown to have a positive contribution to WD responses (Ahmad et al., 2016b; Chen et al., 2017). On the one hand, loss-of-function mutants are sensitive to WD and, on the other hand, overexpressors are tolerant. Similar results were obtained for the rice small vacuole K^+^ channel OsTPKb (Ahmad et al., 2016a). Rice overexpressing lines showed better growth and higher K^+^ uptake capacity than wild-type (WT) plants when exposed to WD conditions. However, the mechanism by which increased OsTPKb activity in small vacuoles led to enhanced root K^+^ uptake and increased K^+^ tissue concentration in rice plants remains uncertain. Overall, the proposed mechanism underlying the phenotypes of OsAKT1, OsHAK1, and OsTPKb overexpressors is that enhanced K^+^ uptake leads to improved plant performance under WD conditions.

Control of stomatal movements is critical for saving water under drought conditions and is largely dependent on K^+^ transport [reviewed by (MacRobbie, 1998; Kwak et al., 2001; Hosy et al., 2003; Roelfsema and Hedrich, 2005; Ward et al., 2009). However, there are not so many studies assessing the impact of altered guard cell K^+^ transport under WD conditions (Nieves-Cordones et al., 2012; Osakabe et al., 2013; Andrés et al., 2014; Jezek and Blatt, 2017; Papanatsiou et al., 2019). The link between guard cell K^+^ transport, stomatal pore size, and WD avoidance is somehow clear but the performance of plants with altered guard cell K^+^ transport under water stress cannot be easily anticipated. In the case of the vacuolar K^+^ channel TPK1, it was shown that loss-of-function *tpk1* plants closed stomata more slowly than WT plants but no phenotype was observed under drought conditions (Gobert et al., 2007; Isner et al., 2018). Regarding K^+^ Shaker-like channels, *GORK* encodes the major voltage-gated outwardly rectifying K^+^ channel in *Arabidopsis* guard cells which allows a rapid release of K^+^ from these cells upon membrane depolarization (Hosy et al., 2003). Consequently, *gork* plants exhibited impaired stomatal closure and consumed more water than WT plants during water stress (Hosy et al., 2003). Different results were obtained in the loss-of-function mutant of the *Arabidopsis* inward-rectifying K^+^ channel AKT1 (*akt1* plants) in which an improved water-deficit response was observed (Nieves-Cordones et al., 2012). This response was explained by enhanced ABA-induced stomatal closure of *akt1* guard cells in stomatal aperture assays with no effect of the *akt1* mutation in the absence of ABA. A similar phenotype was observed for the protein kinase CIPK23 which activates AKT1 (Cheong et al., 2007; Nieves-Cordones et al., 2012; Sadhukhan et al., 2019).

Maintenance of root growth under WD depends on the intensity and duration of the stress. In some species such as maize, it has been shown that root keeps growing in contrast to rice and soybean when a drought treatment is applied (Tanguilig et al., 1987). That leads to higher accumulation of K^+^ (root and shoot), N (root), and P (root) which can help in the plant acclimation to the stress treatment. Auxins are well-known to shape root growth and architecture (Overvoorde et al., 2010). Interestingly, several K^+^ transport systems such as KUP6, TRH1, and AKT1 have been related to root growth traits and to auxin homeostasis (Osakabe et al., 2013; Rigas et al., 2013; Li et al., 2017). For example, it has been proposed that the *Arabidopsis* K^+^ channel AKT1 acts as a sensor that contributes to primary root growth signaling. In low K^+^ media, *akt1* plants did not degrade PIN1 proteins, auxin accumulated in the root tip, and root kept growing in contrast to WT plants (Li et al., 2017). Interestingly, *akt1* roots were also longer than those of WT under WD conditions (Nieves-Cordones et al., 2012) which suggests that AKT1 sensing is of relevance under this stress, too. Thus, the *Arabidopsis* K^+^ channel AKT1 function has been related to root K^+^ uptake (Hirsch et al., 1998), ABA-induced stomatal closure (Nieves-Cordones et al., 2012), and primary root growth (Li et al., 2017).

#### Chloride Membrane Transport

Plants accumulate Cl^–^ to macronutrient levels, around 15–50 mg g^-1^ dry weight (DW), when the external concentration is 1 to 5 mM Cl^–^ (Colmenero-Flores et al., 2019). This range of Cl^–^ concentration is beneficial for the plant and insufficient to cause toxicity. Thus, Cl^–^ represents the dominant inorganic anion in plant cells, with leaf contents that can be similar to those of the macronutrient K^+^ (Franco-Navarro et al., 2016). This accumulation led to better plant performance, the reason why Cl^–^ has been recently defined as a beneficial macronutrient (Franco-Navarro et al., 2016; Raven, 2017; Wege et al., 2017; Colmenero-Flores et al., 2019). It is worth highlighting that field assays using durum wheat have shown that soil Cl^–^ deficiency caused a number of physiological disorders impairing the growth and yield (Schwenke et al., 2015). Therefore, Cl^-^ nutrition should receive particular attention.

Osmoregulation in plant cells occurs mainly in the vacuole through the accumulation of osmolytes, mainly of ionic nature. Vacuoles accumulate high concentrations of Ca^2+^, K^+^, and Na^+^, with Cl^–^ acting as a major counteranion. Chloride is preferentially compartmentalized in the vacuole, where it is normally the most abundant anion. Chloride specifically stimulates plant cell osmolarity and turgor for different reasons: i) Cl^–^ is not metabolized; ii) it stimulates specifically the vacuolar proton-pumping V-type ATP-ase (Sze, 1985), which in turn boosts ion compartmentalization; iii) the stability of water molecules interaction is atypically high in the solvation shell of halogen anions (Marcus, 1991; Kropman and Bakker, 2001), making Cl^–^ very suitable to enhance water retention. In comparison to anionic macronutrients like NO_3_^‒^, SO_4_^2-^, and PO_4_^3-^, Cl^–^ salts specifically promotes higher osmolarity, water content, relative water content, turgor, and leaf succulence (Franco-Navarro et al., 2016). Osmolarity, water accumulation, and turgor are the driving force of plant cell elongation, and Cl^-^ has been proven to specifically stimulates cell elongation in different cell types [reviewed in (Colmenero-Flores et al., 2019)]. Therefore, Cl^–^ stimulation of larger cells with higher osmotic capacity and relative water content give rise to plant tissues with superior water storage capacity (Franco-Navarro et al., 2016). This is a crucial aspect when considering plant acclimation to WD. Interestingly, chloride simultaneously stimulates growth and reduces water consumption, resulting in a clear improvement of water-use efficiency [WUE (Franco-Navarro et al., 2016)]. The beneficial effect of macronutrient Cl^–^ nutrition in maintaining high photosynthesis rates while improving WUE is particularly challenging in C_3_ plants, in which water loss through transpiration is inherent to the process of fixing atmospheric CO_2_. This is because Cl^–^ specifically increases the mesophyll diffusion conductance to CO_2_ (Franco-Navarro et al., 2019; Maron, 2019). This is a consequence, at least in part, of a higher surface area of chloroplasts exposed to the intercellular airspace of mesophyll cells. However, it remains to be understood how Cl^-^ takes part in this issue (**Figure 1**).

Another aspect of Cl^–^ related to water stress avoidance is its role in guard cells movements. They are crucial for stomatal closure and, in turn, to prevent excessive water loss during WD (MacRobbie et al., 1982; Saito and Uozumi, 2019). In this regard, the slow type (S-type) channel SLAC1 (Negi et al., 2008; Vahisalu et al., 2008) and, in a lesser extent, SLAH3 (Geiger et al., 2011) have been shown to mediate Cl^-^ efflux in direct response to ABA stimulation at the plasma membrane of guard cells as a necessary step to trigger stomatal closure. Regarding vacuolar Cl^-^ compartmentalization in guard cells, the quick-type AtALMT9 (De Angeli et al., 2013), and the MATE-type AtDTX33 and AtDTX35 (Zhang et al., 2017) anion channels are required for adequate stomatal functioning. However, it is expected that other yet unidentified transport systems take part in Cl^-^ transport at the plasma membrane and at the tonoplast which are necessary for stomatal movements.

To improve acclimation to WD, ABA increases the accumulation of the most abundant plant electrolytes K^+^ and Cl^–^ within the root by significantly inhibiting the release of these ions into the xylem, but having little effect on their root ion influx (Cram and Pitman, 1972; Cram, 1973; Pitman and Wellfare, 1978) (**Figure 1**). Thus, drought stress and ABA down-regulate the activity and expression of K^+^ and Cl^-^ translocation systems (Gaymard et al., 1998; Roberts, 1998; Kohler and Raschke, 2000; Roberts and Snowman, 2000; Gilliham and Tester, 2005). Recently, Cubero-Font et al. (2016) demonstrated the role of two S-type channels, AtSLAH1 and AtSLAH3, in the regulation of xylem loading of NO_3_^-^ and Cl^-^ in *Arabidopsis thaliana*. The AtSLAH1 protein functions like a molecular switch that regulates the degree of NO_3_^-^ *vs.* Cl^-^ conductance according to environmental cues. Under WD conditions, the expression of the *AtSLAH1* gene is strongly repressed in an ABA-dependent manner (Cubero-Font et al., 2016; Qiu et al., 2016), significantly reducing the Cl^-^ conductance of SLAH3, therefore favoring the retention of Cl^-^ in the root.

#### Root Water Membrane Transport

Water acts as a transport medium for nutrients and metabolites. Leaf transpiration is the main driver of the water flow, from root to leaves, by creating a gradient of water potential between the soil and the atmosphere. When exposed to WD, plants quickly respond to enhance their capacity to uptake water from the soil through the modulation of the hydraulics of root cells and tissues, i.e., their intrinsic permeability to water (Scharwies and Dinneny, 2019). Long-distance water transport occurs through xylem and phloem vessels and WD is well known to produce profound effects on this process (Qaderi et al., 2019). This section is focused on recent findings related to water membrane transport and architecture of roots under WD. Information on long-distance water transport under WD can be found in recent reviews (Sevanto, 2014; Venturas et al., 2017; Scharwies and Dinneny, 2019; Qaderi et al., 2019). Root water uptake mainly occurs by two pathways: cell-to-cell (transcellular and symplastic) and apoplastic (Steudle and Peterson, 1998). In the cell-to-cell pathway, aquaporins are water channel proteins that facilitate water transport across cell membranes and play a major role in the control of water homeostasis inside the plant (Maurel et al., 2015), with contributions up to 80% of the root hydraulic conductivity (*L*p_r_) in *A. thaliana* (Sutka et al., 2011; Li et al., 2015; Rosales et al., 2019). Under stress, *L*p_r_ is mainly modified by aquaporin activity, whose regulation is mostly related to post-translational mechanisms (e.g., phosphorylation) rather than changes in gene expression (Aroca et al., 2012; Li et al., 2015; Sanchez-Romera et al., 2018). When soil water potential strongly decreases, water leaves the cells and turgor pressure falls rapidly. In response to severe WD, roots quickly reduce the aquaporin activity and *L*p_r_ to avoid the root-to-soil water flow (Sutka et al., 2011; Hachez et al., 2012; Rosales et al., 2019). However, under mild drought stress, some plant species are able to enhance the *L*p_r_ and aquaporin activity as a response mechanism mediated by ABA to promote water uptake and transport, when the difference of water potential between roots and soil has already been readjusted (Siemens and Zwiazek, 2004; Aroca et al., 2006; Rosales et al., 2019). When drought stress is prolonged, plants have shown to respond by developing root apoplastic barriers as suberization (Vandeleur et al., 2009; Henry et al., 2012), and by even inducing the xylem cavitation that results in the formation of embolism and disruption of the transpiration stream (Qaderi et al., 2019).

In leaves, drought stress alters the water transport through the xylem (e.g., petiole and veins) and, specially, out of the xylem in the bundle sheath and mesophyll (Scoffoni et al., 2011; Buckley, 2015; Maurel et al., 2015). ABA is a key hormone that induces the stomatal closure in response to WD by controlling the movement of ions and water across the guard cell plasma membrane and, finally, the transpiration and plant water status (Roelfsema and Hedrich, 2005; Kim et al., 2010). Aquaporins control the water transport in guard cells and its regulation has been linked to the stomatal regulation in higher plants (Maurel et al., 2016). Recently, *At*PIP2;1 has been found to be activated by ABA through OST1-mediated phosphorylation of a specific cytosolic site (Ser121), being essential for ABA-induced stomatal closure (Grondin et al., 2015). Furthermore, leaf hydraulics has been demonstrated to decrease under drought stress, by the downregulation of aquaporins in bundle sheath cells and leaf xylem embolism (Hose et al., 2000; Johnson et al., 2009; Shatil-Cohen et al., 2011; Pou et al., 2013; Sade et al., 2015).

In the longer-term, plants also regulate water uptake in response to WD by adjusting the root architecture, which results from root growth and branching determining the ability of roots to explore the soil (Lynch, 2013). Water and nutrients are usually distributed heterogeneously in the soil and exert deep effects on both root hydraulics and architecture, being central for plant’s acclimation to drought by optimization of water uptake. A common response to severe drought in higher plants is the inhibition of plant growth and development, which have been related to an accumulation of ABA in both roots and shoots (Wilkinson et al., 2012; Comas et al., 2013; Claeys et al., 2014; Rosales et al., 2019), whereas root growth stimulatory responses under mild WD have been also reported (Henry et al., 2011; Dowd et al., 2019; Rosales et al., 2019). Recently, a fine analysis of root system architecture in *A. thaliana*, by using different ABA mutants and applying low ABA concentrations. Recently, a fine analysis of root system architecture in *A. thaliana*, by using different ABA mutants and applying low ABA concentrations, have revealed a complex response to WD that resulted to be coordinated by ABA, showing different sensitivity depending on root age and branching level (Rosales et al., 2019). ABA has been identified as a key component of several major adaptive responses to local WD (Scharwies and Dinneny, 2019). In primary roots, the local activation of ABA signaling in the root cortex of the elongation zone regulates their hydrotropic response of growth toward the water in *Arabidopsis* (Dietrich et al., 2017). In cereals, the transient WD at the root tip resulted in a local inhibition of lateral root formation, a phenomenon termed xerobranching, which is dependent on PYRABACTIN RESISTANCE/PYR-LIKE ABA signaling pathway (Orman-Ligeza et al., 2018). Finally, ABA and auxin signaling pathways have been shown to control hydropatterning, positioning preferentially the lateral roots toward higher water availability (Bao et al., 2014; Orosa-Puente et al., 2018; Robbins and Dinneny, 2018).

### Improving Crop Yield Under Drought in the Field

**Figure 2:**
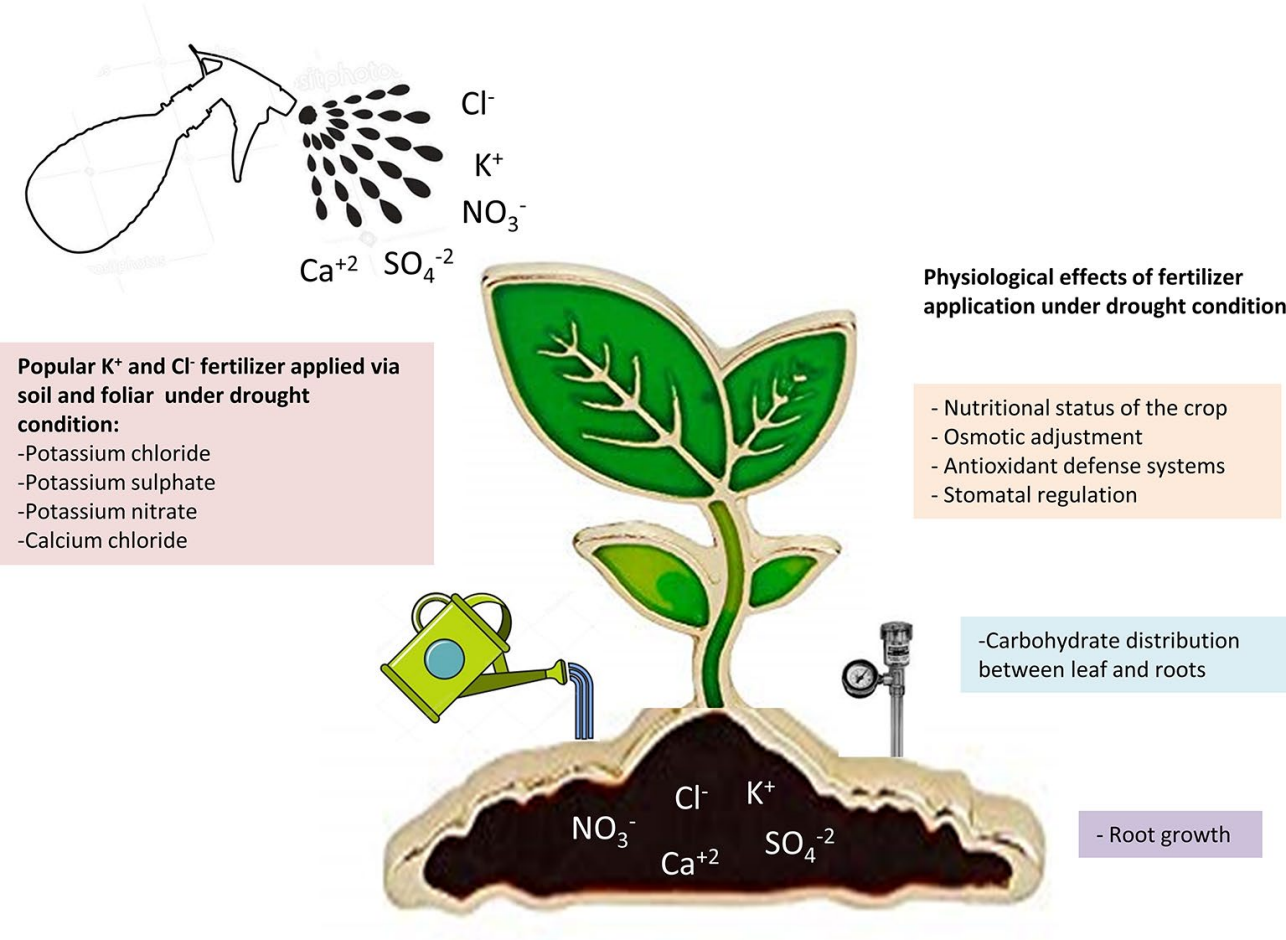
Summary of the main K^+^ and Cl^-^ fertilizers applied to crops and their beneficial effects under soil water deficit.

Current precision agriculture is based on the combined use of water and fertilizers as a strategy to optimize inputs, maximize performance, and improve the quality of the crops. In arid and semi-arid regions, where the precipitation does not exceed 300 mm annually, the sustainability of the crops is strongly linked to the correct management of fertigation (**Figure 2**). In the current scenario of climate change, drought periods are becoming more frequent, forcing the growers to irrigate below the water requirements of the crops (Chai et al., 2015). Numerous studies have shown that the appropriate supply of nutrients to the crops can help plants withstand WD (Garcia-Sanchez et al., 2016). Thus, fertilization with main macronutrients (N-P-K) can be a general strategy to mitigate the effects of drought. Unlike K^+^, nitrogen (N) and phosphorus (P) are metabolized in the plant and their contribution to cell osmotic potential as inorganic salts is lower than that of K^+^. However, they are a part of organic molecules with osmoregulation and antioxidant capacity. Thus, it is not surprising that additional inputs of N and P have a beneficial effect on plants under drought stress (Tariq et al., 2018; Mahpara et al., 2019). With respect to K^+^, in cotton (Pervez et al., 2004) and citrus crops (Gimeno et al., 2014), it has been observed that an adequate level of K^+^ in the irrigation water increases their tolerance to drought. Recently, it has also been observed that K^+^ regulates the processes of photo-assimilation and translocation of carbohydrates in cotton plants, favoring their acclimation to drought stress (Zahoor et al., 2017). Regarding Cl^–^ fertilization, substantial responses to Cl^-^ containing fertilizers have been reported for different crops in many parts of the world (Xu et al., 1999; Chen et al., 2010; Colmenero-Flores et al., 2019). However, these studies do not clarify to what extent plant yield enhancement was due to the accompanying cations, or whether other anions could replace Cl^–^ in such growth-promoting effect. It has been recently proven that a number of physiological disorders impairing the growth and yield of a relevant crop, durum wheat, are specifically due to soil Cl^–^ deficiency under field conditions (Schwenke et al., 2015). These and other crop species are expected to be specifically favored by Cl^–^ fertilization.

## Conclusions and Perspectives

In the present mini-review, we have summarized the recent advances related to K^+^, Cl^-^, and water membrane transport under WD conditions. The promising results obtained so far strongly encourage the study of transport systems mediating in K^+^, Cl^-^, water fluxes in plants and should be regarded as interesting biotechnological targets to breed drought-tolerant crops. These transport systems usually have multiple roles within the plant. So, additional work is required in assessing the potential benefit of improving K^+^, Cl^-^, and water transport in a certain cellular type or tissue (i.e., guard cells, mesophyll cells, and xylem parenchyma cells) to better understand drought resistance mechanisms. Another scientific challenge relies on the cross-talk between auxins and ABA during WD. They have an outstanding importance on plant cell growth rates, in particular for root cells, and on K^+^, Cl^-^, and water transport. So, further insights into how these hormones regulate K^+^, Cl^-^, and water transport and in how these processes are integrated into drought-tolerant genotypes are required. In the field, fertigation is the main solution adopted by farmers to improve plant nutrient status under WD. However, we expect that new strategies can emerge from the aforementioned proposed studies leading to increased productivity of agriculture in the XXI century.

## Author Contributions

JC-F and MR conceived the idea. FG-S and JP-P wrote “improving crop…” section and made figure 2. MN-C and FR wrote “potassium membrane transport” section and made figure 1. JC-F wrote “chloride membrane transport” section. MR wrote “root membrane water transport”. MN-C wrote the rest of the text and assembled all sections.

## Funding

This work was supported by Grant 20806/PI/18 from Fundación Séneca de la Región de Murcia (to FR) and AGL2015‐74011‐JIN (to MN-C) from the Spanish Ministry of Economy and Competitiveness. MN-C and JP-P are recipients of a Ramón y Cajal Fellowship (RyC-2017-21924 and RYC-2015-17726, respectively) from Spanish Ministry of Economy and Competitiveness, Spain. This work was supported by the Spanish Ministry of Science Innovation and Universities-FEDER grant RTI2018-094460-B-I00 and by the Spanish National Research Council Project CSIC-201940E039 (to JC-F and MR). This work was also supported by the Network of Excellence BIO2016‐81957‐REDT funded by the Spanish Ministry of Economy and Competitiveness.

## Conflict of Interest

The authors declare that the research was conducted in the absence of any commercial or financial relationships that could be construed as a potential conflict of interest.
